# ABA Suppresses *Botrytis cinerea* Elicited NO Production in Tomato to Influence H_2_O_2_ Generation and Increase Host Susceptibility

**DOI:** 10.3389/fpls.2016.00709

**Published:** 2016-05-25

**Authors:** Anushen Sivakumaran, Aderemi Akinyemi, Julian Mandon, Simona M. Cristescu, Michael A. Hall, Frans J. M. Harren, Luis A. J. Mur

**Affiliations:** ^1^Molecular Plant Pathology Group, Institute of Biological, Environmental and Rural Sciences, Aberystwyth UniversityAberystwyth, UK; ^2^Life Science Trace Gas Facility, Molecular and Laser Physics, Institute for Molecules and Materials, Radboud UniversityNijmegen, Netherlands

**Keywords:** nitric oxide, reactive oxygen species, ethylene, ABA, *sitiens*, *Botrytis cinerea*

## Abstract

Abscisic acid (ABA) production has emerged a susceptibility factor in plant-pathogen interactions. This work examined the interaction of ABA with nitric oxide (NO) in tomato following challenge with the ABA-synthesizing pathogen, *Botrytis cinerea.* Trace gas detection using a quantum cascade laser detected NO production within minutes of challenge with *B. cinerea* whilst photoacoustic laser detection detected ethylene production – an established mediator of defense against this pathogen – occurring after 6 h. Application of the NO generation inhibitor *N*-Nitro-L-arginine methyl ester (L-NAME) suppressed both NO and ethylene production and resistance against *B. cinerea*. The tomato mutant *sitiens* fails to accumulate ABA, shows increased resistance to *B. cinerea* and we noted exhibited elevated NO and ethylene production. Exogenous application of L-NAME or ABA reduced NO production in *sitiens* and reduced resistance to *B. cinerea.* Increased resistance to *B. cinerea* in *sitiens* have previously been linked to increased reactive oxygen species (ROS) generation but this was reduced in both L-NAME and ABA-treated *sitiens.* Taken together, our data suggests that ABA can decreases resistance to *B. cinerea* via reduction of NO production which also suppresses both ROS and ethylene production.

## Introduction

The outcome of pathogen interactions with plants is governed by multiple events (reviewed by Jones and Dangl, 2006). Initial contact involves host detection of pathogen-associated molecular patterns [PAMPs, microbial-associated molecular patterns; also referred to as (MAMPs)] to initiate PAMPs-triggered immunity (PTI). Pathogens have evolved a range of effectors which can suppress PTI, some of which may be recognized by the host–classically by Resistance (*R*) genes – to initiate effector-triggered immunity (ETI) which can lead to the form of programmed cell death known as the hypersensitive response (HR; Mur et al., 2008). Superimposed on these interactions are the various pathogen-derived toxins, enzymes, and host modifying proteins that can drive symptom development. Based on disease symptoms it is possible to crudely classify pathogens into biotrophs (parasitizing living host tissue) or necrotrophs (predating death host tissue) or those exhibiting combinations of both lifestyles – hemibiotrophs (Schulze-Lefert and Panstruga, 2003; Oliver and Ipcho, 2004; Oliver and Solomon, 2010).

Pathogen-associated molecular patterns-triggered immunity and ETI-linked defense gene expression is co-ordinated by a large number of hormones (Robert-Seilaniantz et al., 2007). Traditionally, salicylic acid (SA) mediated defenses have been linked to defense against biotrophs and jasmonates (JA)/ethylene (Et) against necrotrophs (Oliver and Solomon, 2010) although this represents a considerable over-simplification (Robert-Seilaniantz et al., 2011). Nitric oxide (NO) should also be considered as a major defense initiator (Mur et al., 2013). Thus, NO has been shown to be rapidly generated in plants following challenge with biotrophic and necrotrophic pathogens (Delledonne et al., 1998; Foissner et al., 2000; Mur et al., 2005, 2012; Prats et al., 2005; Besson-Bard et al., 2008). Despite many mechanisms of NO generation being proposed, it appears that under aerobic conditions NO is mostly produced by cytosolic nitrate reductase (NR) acting on ${\mathrm{NO}}_{2}^{–}$ as a nitrite reductase (Gupta et al., 2011). NO production has been linked to PTI, the HR and the formation of defense associated cell-wall appositions (Zeidler et al., 2004; Prats et al., 2005; Mur et al., 2008). Further, the importance of NO to plant defense has been shown through the use of chemical inhibitors, transgenic lines, or differential N fertilizer regimes to modify NO production and impact on the degree of resistance (Delledonne et al., 1998; Boccara et al., 2005; Mur et al., 2012; Gupta et al., 2013). The mechanisms through which NO impacts on defense signaling cascades have also been extensively examined, with reversible *S-*nitrosylation of proteins and irreversible tyrosine nitration of key components emerging as important regulatory events (Cecconi et al., 2009; Leitner et al., 2009). Notable examples of the role of *S-*nitrosylation in defense are the modification of the vital SA signaling component NON-EXPRESSOR OF PATHOGENESIS-RELATED PROTEIN1 (NPR1; Tada et al., 2008) and the reactive oxygen species (ROS) generating complex NADPH oxidase, AtRBOHD (Yun et al., 2011). Further, there is clear evidence that NO, ROS, and SA pathways work together to establish a systemic acquired resistance (SAR) that can be exhibited by the whole plants following the localized initiation of resistance (Wang et al., 2014). Such examples demonstrate the close interactive role of NO with other signaling pathways which also include JA and Et (Mur et al., 2013).

Abscisic acid (ABA) is classically associated with plant responses to abiotic stress particularly drought (Shinozaki and Yamaguchi Shinozaki, 1996) but is now well recognized to also play roles in plant responses to pathogens (Cao et al., 2011). However, rather than aiding resistance, ABA, appears to be mostly contributing to susceptibility. Thus, exogenous application of ABA increased the susceptibility of *Arabidopsis* to *Pseudomonas syringae* pv. *tomato* (Fan et al., 2009) or of rice to the rice blast pathogen *Magnaporthe grisea*; in this latter case even with avirulent strains (Jiang et al., 2010). Correspondingly, *Arabidopsis* ABA biosynthetic mutants exhibit enhanced resistance to *P. syringae* (de Torres-Zabala et al., 2007; Goritschnig et al., 2008). Considering mechanisms through which ABA antagonisms act, one mechanism appear to perturbation of SA signaling. Some key studies have shown that ABA suppresses expression of the SA biosynthetic gene *ISOCHORISMATE SYNTHASE1* (*ICS1*) in *Arabidopsis* (Yasuda et al., 2008) and (e.g.) *NPR1* in rice (Jiang et al., 2010). Other workers have shown a reciprocal antagonism with the increased disease resistance in the *Arabidopsis* mutant *cpr22* at least partially linked to SA-mediated perturbation of ABA signaling (Moeder et al., 2010; Mosher et al., 2010). However, ABA impacts are not only associated with SA-mediated defenses – which are mostly deployed against biotrophs. ABA effects can also be seen in initiating ROS generation with latter contributing to ABA signaling (Pei et al., 2000). Similarly, ABA increase NO generation which can increase the activity of antioxidant enzymes (Zhou et al., 2005). Such observations suggest a subtle interaction between ABA, ROS, and NO.

Abscisic acid treatment could also compromise the resistance of *Arabidopsis* to the necrotroph *B. cinerea* (AbuQamar et al., 2006). Much work on the interaction of ABA and *Botrytis* has focused on the tomato mutant *sitiens* (Asselbergh et al., 2007). *sitiens* is deficient in ABA production through a mutation in the biosynthetic ABA-aldehyde oxidase (Harrison et al., 2011). *sitiens* exhibits considerably increased resistance to *B. cinerea* and this has been variously linked to increased SA effects (Audenaert et al., 2002) and/or epidermal hydrogen peroxide generation with cell wall modifications (Asselbergh et al., 2007). A similar link between ABA and suppression of ROS during attack by *B. cinerea* has been made in *Arabidopsis* (L’Haridon et al., 2011). Given such observations it is unsurprising that *B. cinerea* strains can encode genes for ABA biosynthesis (Gong et al., 2014).

In this current study, we examine the interaction between ABA and NO generation in tomato. Through the use of pharmacological inhibitors of NO generation we demonstrate the importance of NO to tomato defense against *B. cinerea.* Exploiting online, *in planta* measurements, we show a greatly elevated NO generation in the ABA deficient mutant *sitiens*. Suppression of NO production in *sitiens*, with NO generation inhibitors or the exogenous application of ABA, also compromised the increased resistance in this mutant to *B. cinerea*. Staining ROS production using 3,3′-diaminobenzidine (DAB) suggested that the levels were influenced by the rates of NO generation. We propose that ABA-influenced antagonism of NO generation could be an important mechanism through which pathogens can suppress defenses to establish compatible interactions with their host.

## Material and Methods

### Tomato Plant Growth Conditions

Tomato genotypes, cv. Ailsa Craig and *sitiens* were obtained from the Tomato Genomics Resource Centre (TGRC) at UC, Davis USA^1^. Tomato plants were cultivated in Levington Universal compost in 10 cm diameter plastic pots. Plants were maintained in Conviron (Controlled Environments Ltd, UK) growth rooms at 24°C with a light intensity of 110 μmol/m/s and a 12 h photoperiod for 4 weeks. At 4 weeks *sitiens* plants were considerably smaller than cv. Ailsa Craig but exhibited no evidence of any other defects (e.g., wilting) that were visible to the naked eye. Plants used for NO and Et measurements were transported to Radboud University (The Netherlands) via road and car ferry by the authors. Plants were then kept at Radboud University for 2 days under identical growth conditions to those in Aberystwyth to allow plant physiology to normalize prior to gas measurements being attempted.

### *Botrytis cinerea* Culture and Storage

*Botrytis cinerea* (strain IMI 169558) was maintained on potato dextrose agar (PDA) in an inverted position in a Gallenkamp illuminated cooled incubator maintained at 20°C with a 12 h photoperiod. PDA (Potato extract 4.0 g L^-1^; Glucose 20.0 g L^-1^; Agar 15.0 g L^-1^) was prepared according to manufacturer’s instructions.

Conidia were harvested from the plate surface by flooding the plate with a carrier media of potato dextrose broth (PDB) and dislodging the conidia with an L-shaped glass rod. The density of conidia in the suspension was determined using a Neubauer Haemocytometer and the solution was diluted accordingly to 1 × 10^5^ conidia mL^-1^ to provide a 30 mL standardized infection solution.

Four-week old tomato plants were inoculated with *B. cinerea* in one of two methods. Where trace gas detection (NO or Et) was being measured, the conidial suspension was sprayed on to plants to run-off. In all other occasions, inoculation involved application of 10 μL drops of spore solution, pipetted directly onto the adaxial leaf surface. Inoculation was made to the first four true leaves (excluding cotyledons), with seven drops of solution being applied to three leaflets per leaf with a total of 84 drops per plant.

### Chemical Treatment of Tomato Plants

In wild type cv. Ailsa Craig plants, chemicals that would alter NO production were applied through the excised petioles of branches so that the entire compound leaf was treated. Selected compound leaves represented the oldest true leaves of 4-week-old plants. Excised branches were placed in 100 mL beakers with 50 mL of either 0.1 mM sodium nitroprusside (SNP, an NO donor) or 5 mM *N*-Nitro-L-arginine methyl ester (L-NAME; an inhibitor of NO generation) or *N*-Nitro-D-arginine methyl ester (D-NAME; a biologically inactive isomer of L-NAME) for 24 h prior to infecting the leaves with *B. cinerea.* Excised branches in beakers were maintained at 24°C with a light intensity of 110 μmol/m/s and a 12 h photoperiod before and after spot-inoculation with *B. cinerea.*

Chemicals could not be applied to *sitiens* plants through cut petioles as this led to rapid tissue collapse of the leaves. Instead, chemicals were applied to intact 4-week-old *sitiens* plants as a foliar spray which included 0.2% Silwet (v/v) as wetting agent. Although this may not have allowed accurate estimations of applied dose to be determined it proved to be the only means (in our hands) to apply chemicals and maintain *sitiens* leaf viability.

### Estimating Fungal Biomass by Quantitative PCR (qPCR)

DNA was extracted from eight leaf samples with single inoculations of *B. cinerea* using a DNeasy Mini Kit (Qiagen, UK) following manufacturer’s instructions. DNA samples were diluted to 1 ng μL^-1^ ultrapure H_2_O. Samples (25 μL) were prepared by mixing 10 μL DNA solution with 16 μL SYBR^TM^ Green Mastermix (Applied Biosystems, UK) and primers (300 nM) for *Arabidopsis* (iASK1: CTTATCGGATTTCTCTATGTTTGGC; iASK2: GAGCTCCTGTTTATTTAACTTGTACATACC) to generate an 131 bp amplicon and *B. cinerea* (CG11: AGCCTTATGTCCCTTCCCTTG; CG12: GAAGAGAAATGGAAAATGGTGAG) to generate a 58 bp amplicon (Gachon and Saindrenan, 2004). PCR used a Bio-Rad ABI7300 thermocycler amplifying using the following conditions: 15 min at 95°C followed by 50 cycles of 95°C for 15 s, 58°C for 30 s and 72°C for 1 min. This was followed by a dissociation (melting curve), according to the software procedure. Serial dilutions of pure genomic DNA from each species were used to trace a calibration curve, which was used to quantify plant and fungal DNA in each sample. Results were expressed as the CG11/iASK ratio of mock to inoculated samples.

### Nitric Oxide (NO) Measurements Using a Quantum Cascade Laser-Based Sensor

The configuration of the quantum cascade laser (QCL)-based sensor for NO detection is detailed elsewhere (Cristescu et al., 2008). Briefly, the QCL emitting infrared light around 1900 cm^-1^ that passes through an absorption multi-pass cell, in which the airline enters transporting the NO released by a single inoculated rosette within a glass cuvette (∼500 mL volume). The NO production is directly detected by measuring the attenuation of the laser intensity due to the NO absorption in the cell. Infected plants were placed in glass cuvette and flushed with air at a controlled continuous flow rate of 1 L h^-1^. Multiple cuvettes could be monitored in sequence, each being measured for ∼13 min. Each experiment was repeated to give similar results and the outcomes of one representative experiment are shown.

### Ethylene Measurements Using Photoacoustic Laser Spectroscopy

Ethylene production was monitored in real-time using a gas flow-through in-line system fitted with a photoacoustic laser-based ethylene detector (ETD-300, Sensor Sense BV) able to detect on-line 300 parts per trillion volume of ethylene within 5 s (Cristescu et al., 2008). Gas was regulated by an automated valve control box (VC-6, Sensor Sense B.V) and ethylene emanation from a tomato plant within a glass cuvette (254 mL volume) was alternately monitored for 15 min (5 s per acquisition point), at a controlled continuous flow rate of 1.5 L h^-1^ by flushing with air and preventing accumulation induced effects. KOH and CaCl_2_ scrubbers were incorporated into the system to remove CO_2_ and H_2_O, respectively. Each experiment was repeated to give similar results and the outcomes of one representative experiment are shown.

### Visualization and Quantification of H_2_O_2_ Generation

After sampling, the excised leaf disks were immersed in an aqueous solution of 1 mg ml^-1^ DAB (pH 3.8) and incubated at room temperature for 4 h. The leaves were removed from the DAB solution and fixed and cleared in absolute ethanol. The samples were scanned using a flat-bed scanner and the intensity of staining was quantified using Image-Quant TL, Software (GE Healthcare Life Sciences, UK).

### Statistical Analyses

Data were subjected to analysis of variance using Minitab v.14 (Minitab Ltd, Coventry, UK), after which residual plots were inspected to confirm data conformed to normality. Comparisons of data points from different treatments with controls were performed using Tukey multiple pairwise comparison test. Timecourse data were compared using repeated measures anova. Differences with *P* < 0.05 were considered significant.

## Results

### NO Production Is Tomato Is Required for Resistance to *B. cinerea*

**FIGURE 1:**
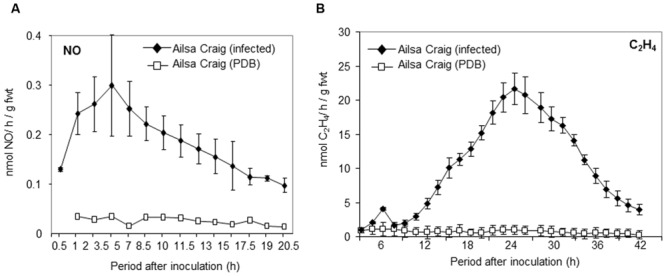
**Nitric oxide (NO) and ethylene production in tomato cultivar Ailsa Craig challenged with *Botrytis cinerea.* (A)** NO and **(B)** ethylene were measured, following spraying whole 4 weeks old tomato Ailsa Craig plants with **(◆)** conidial suspensions of *B. cinerea* in potato dextrose broth (PDB) or **(□)** PDB alone, using, respectively, on-line quantum cascade laser (QCL) detection or photoacoustic laser detection. The presented data are the result of measurements of two *B. cinerea* challenged tomato plants and two PDB challenged tomato plants for each gas. The error bars therefore represent the range of results around a mean (*n* = 2).

We have demonstrated that NO contributes both to ethylene production and resistance to *B. cinerea* in *Arabidopsis* (Mur et al., 2012). To investigate the role of NO in tomato challenged with *B. cinerea*, the production of NO was measured using a QCL-based approach (**Figure 1A**). NO was rapidly produced in cv. Ailsa Craig following infection with *B. cinerea* even before it was possible – due to technical limitations – to take the first measurement at 0.5 h after inoculation. NO production was in slow decline over the subsequent 24 h period. This was specific to pathogen attack as application of PDB, used to resuspend the fungal conidia, failed to elicit increased NO production (**Figure 1A**). Ethylene production was also elicited following challenge with *B. cinerea* but maximum rates of production were achieved some 6 h after that of NO (**Figure 1B**).

**FIGURE 2:**
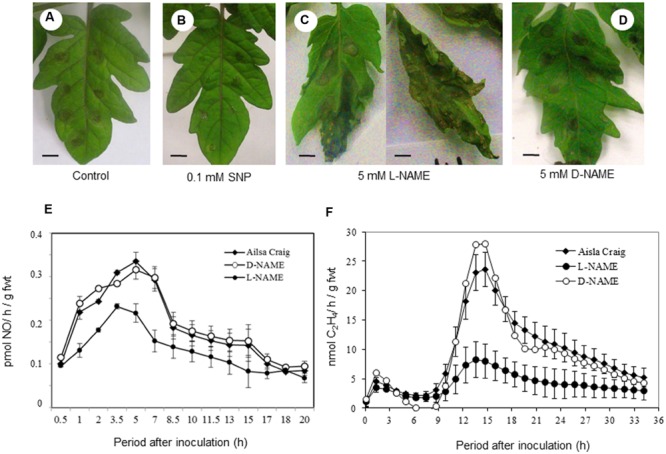
**Sodium nitroprusside (SNP) and *N*-Nitro-l-arginine methyl ester L-NAME) effects on *B. cinerea* lesion development in tomato.** Excised compound leaves from 4 weeks old Ailsa Craig plants were placed in 100 mL beakers with 50 mL of either **(A)** water, **(B)** 0.1 mM SNP (an NO donor) or **(C)** 5 mM *N*-Nitro-l-arginine methyl ester (L-NAME; an inhibitor of NO generation) or **(D)** *N*-Nitro-d-arginine methyl ester (D-NAME; a biologically inactive isomer of L-NAME) for 24 h prior to infecting the leaves with drops of conidial suspensions of *B. cinerea.* Excised compound leaves were maintained at 24°C in beakers with a light intensity of 110 μmol/m/s and a 12 h photoperiod when they were spot-inoculated with *B. cinerea.* The lesions were photographed at 72 h after challenge with *B. cinerea.* Bars = 1 cm. **(E)** NO and **(F)** ethylene were measured in *B. cinerea* infected compound leaves in **(◆)** water-fed Ailsa Craig plants and plant treated with either 5 mM L-NAME **(●)** or 5 mM D-NAME **(○)**. NO and Et were measured using QCL detection or photoacoustic laser detection, respectively. The presented data are the result of measurements of two *B. cinerea* challenged compound leaves for each gas. The error bars therefore represent the range of results around a mean (*n* = 2).

To investigate the role of NO in contributing to resistance to *B. cinerea* experiments were undertaken when a NO donor (SNP) or an inhibitor of NO production (L-NAME) were applied to tomato plants. Thus, 0.1 mM SNP was applied to cut petioles of plants and after 24 h, inoculated with *B. cinerea* with lesion development assessed over a subsequent 72 h period. The developing *B. cinerea* lesions appeared to be smaller and more defined that those forming on controls at 72 h (compare **Figures 2A,B**). Conversely, if the 5 mM L-NAME – was applied to tomato leaves, *B. cinerea* lesions were highly variable in phenotype but were also larger than in control infections and occasionally coalesced to cover the entire leaf (**Figure 2C**). If 5 mM D-NAME – an inactive isomer of L-NAME – was applied to the leaves, the *B. cinerea* lesions appeared to be more similar to those forming in control cv. Ailsa Craig plants (compare **Figures 2D** with **2A**).

Measuring NO production in cv. Ailsa Craig and L-NAME-NAME fed plants suggested that the NO production was significantly (*P* < 0.001) reduced only in L-NAME-treated plants (**Figure 2E**). Correspondingly, laser photoacoustic detection indicated that maximal ethylene production was seen following 9 hpi (hour after infection) was significantly (*P* < 0.001) reduced with L-NAME treatment (**Figure 2F**). Taken together, these data suggest that NO, which may trigger ethylene production, contributes to tomato defense against *B. cinerea.*

### The ABA Mutant Sitiens Displays High Level NO Production

**FIGURE 3:**
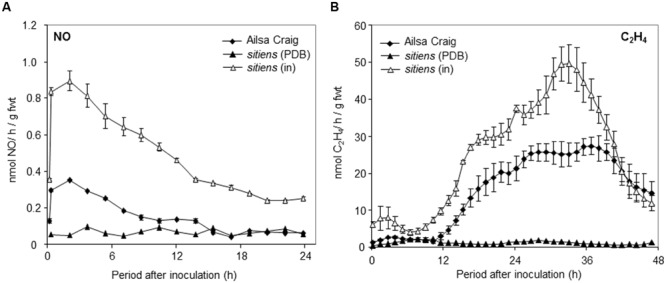
**Nitric oxide and ethylene production in *sitiens* tomato plants challenged with *B. cinerea.* (A)** NO and **(B)** ethylene were measured, following spraying whole 4 weeks old tomato **(◆)** cv. Ailsa Craig or **(Δ)** *sitiens* plants with conidial suspensions of *B. cinerea* in PDB or **(▲)** *sitiens* plants with PDB alone using on-line QCL or photoacoustic laser for NO and ethylene detection, respectively. The presented data are the result of measurements of two *B. cinerea* challenged tomato plants and two PDB challenged tomato plants for each gas. The error bars therefore represent the range of results around a mean (*n* = 2).

We subsequently sought to test if NO played a role in the elevated resistance to *B. cinerea* which is exhibited by the ABA-biosynthetic *sitiens* tomato mutant. Measuring *B. cinerea* elicited NO in *sitiens* showed >2-fold greater rates of production compared to cv. Ailsa Craig (**Figure 3A**). The levels of NO produced in unchallenged *sitiens* plants did not significantly differ from those of cv. Ailsa Craig. *B. cinerea-*induced production of ethylene was also elevated in *sitiens* plants (**Figure 3B**).

**FIGURE 4:**
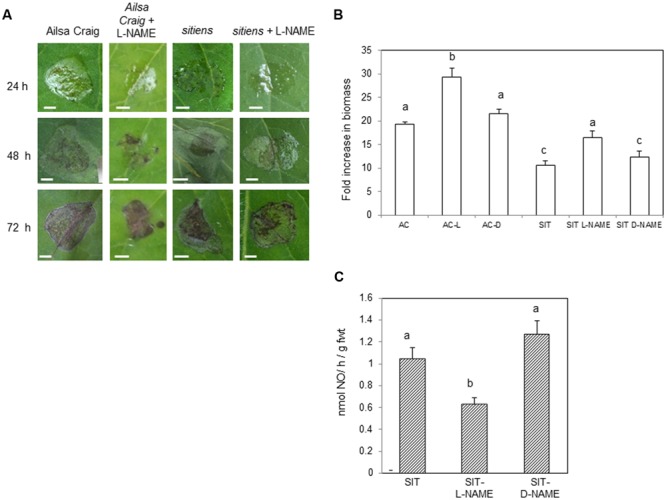
**The effect of reduced NO production on *Botrytis cinerea* infections of *sitiens.* (A)** Representative *B. cinerea* lesion phenotypes in cv. Ailsa Craig and *sitiens* sprayed with either water or 5 mM Nitro-L-arginine methyl ester (L-NAME); an inhibitor of NO production. Note the patterns of water-soaking (tissue collapse) and necrosis (black). Bars = 0.5 cm. **(B)**. *B. cinerea* fungal DNA content in 1 cm^2^ diameter cored lesions isolated from 4 weeks old control cv. Ailsa Craig (AC), *sitiens* (SIT) sprayed 1 h prior to spot-inoculation with *B. cinerea*, sprayed with either L-NAME (L) or D-NAME (D; an inactive L-NAME isomer, 5 mM Nitro-d-arginine methyl ester) sampled at 72 h post-infection. Results are expressed as fold increase in biomass compared to *t* = 0 samples. Mean measurements (*n* = 5 cores) ±SE following inoculation within *B. cinerea* are given. Significant differences are indicated with letters. **(C)** NO production measured at 6 h (peak NO production; see **Figure 1A**) following spraying conidial suspensions of *B. cinerea* of whole 4 weeks old tomato *sitiens* plants. Three treatments were assessed; untreated *sitiens* plants (SIT), plants sprayed 24 h prior to inoculation with *B. cinerea* with either L-NAME (SIT-L-NAME) or D-NAME (SIT-D-NAME). Mean NO production was measured in three plants per treatment using a QCL system. Significant differences are indicated with letters.

In order to establish the importance of increased NO in *sitiens* elevated resistance, cv. Ailsa Craig and *sitiens* plants were sprayed with L-NAME. The resulting lesions appeared to be form less quickly in L-NAME sprayed plants (**Figure 4A**). Lesions forming on D-NAME treated plants did not appear different to the water treated control on either genotype (data not shown). To quantify the differences in lesion formation, *B. cinerea* fungal biomass was measured in lesions developing in cv. Ailsa Craig, *sitiens* and *sitiens* plants sprayed with either L-NAME or D-NAME at 72 hpi (**Figure 4B**). This indicated reduced fungal development in *sitiens* which was consistent with the elevated resistance seen in this line. However, fungal biomass was significantly increased following L-NAME but not D-NAME, implicating NO as a component of cv. Ailsa Craig and; crucially, *sitiens* defense against *B. cinerea.* To confirm that that L-NAME was having an effect on NO production in *sitiens*, this was assessed at 6 hpi with *B. cinerea* in a parallel experiment. This a timepoint which has previously been indicated as the displaying maximal rates of NO production (**Figures 1A** and **3A**). Spraying *sitiens* plants with L-NAME significantly (*P* < 0.01) decreased *B. cinerea* induced NO production compared to chemically untreated *sitiens* or D-NAME treated controls (**Figure 4C**). Logistical constraints prevented the effects of L/D-NAME on NO production to be assessed in cv. Ailsa Craig.

### ABA Suppresses *B. cinerea* NO and ROS Production

**FIGURE 5:**
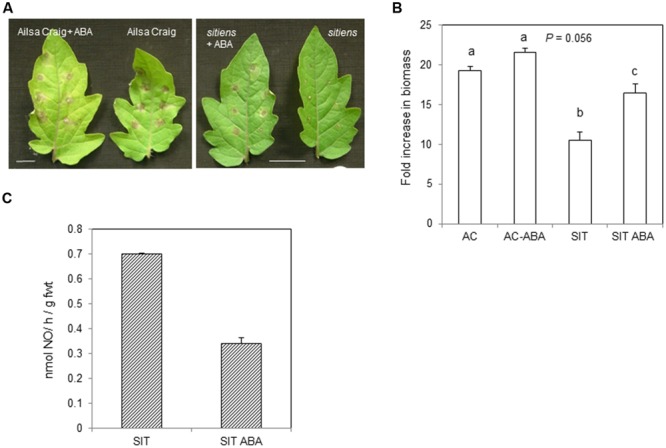
**Nitric oxide production in *B. cinerea* challenged *sitiens* treated with abscisic acid (ABA). (A)** cv. Ailsa Craig or *sitiens* plants were sprayed with either 100 mL ABA in 0.2% Silwet (v/v) or with water [+0.2% Silwet (v/v)] and then multiply spot-inoculated with *B. cinerea* after 1 h. Lesion phenotypes were imaged at 72 hpi. Bars = 1 cm. **(B)** *B. cinerea* fungal DNA in cv. Ailsa Craig (AC), *sitiens* (SIT) sprayed with either 100 mL ABA in 0.2% Silwet (v/v) or with water [+0.2% Silwet (v/v)]1 h prior to spot-inoculation with *B. cinerea.* 1 cm^2^ diameter cored lesions were sampled at 72 h post-infection. Results are expressed as fold increase in biomass compared to *t* = 0 samples. Mean measurements (*n* = 5 cores) ±SE following inoculation within *B. cinerea* are given. Significant differences are indicated with letters. **(C)** NO was measured, following spraying whole 4 weeks old *sitiens* tomato plants with conidial suspensions of *B. cinerea. sitiens* plants were either sprayed with water (SIT) or 100 mM ABA (SIT ABA, 2 repetitions) 1 h prior to challenge with *B. cinerea*. Data are presented at 6 hpi when maximal NO production has been demonstrated (see **Figures 1** and **2**). The error bar represent the range of results around a mean (*n* = 2).

As *sitiens* is deficient in ABA, it was hypothesized that exogenous application of ABA could reduce NO production. Thus, *sitiens* plants as well as cv. Ailsa Craig were sprayed with 100 mM ABA and then after 1 h, we challenged with *B. cinerea.* Examining the effect of ABA treatment on lesion development in *sitiens* suggested that lesion sizes at 72 hpi tended to be greater compared to untreated controls. The phenotypic effects of ABA on cv. Ailsa Craig were more difficult to assess visually (**Figure 5A**). This slight effect of ABA on *B. cinerea* infected on of cv. Ailsa Craig was also suggested from estimations of fungal biomass at 72 hpi (**Figure 5B**). Although there was a trend toward increased fungal development with ABA treatment differences were not significant compared to controls. This was in contrast to *sitiens* where the application of ABA significantly increased fungal biomass suggesting that resistance was being reduced.

The impact on NO on ABA in *sitiens* was measured using the QCL-based system (**Figure 5C**). Logistical constraints limited the number of samples that could be measured so only the effect of ABA on *sitiens* was determined following challenge with *B. cinerea*. NO production in the control plant was in line with that previously observed (e.g., **Figure 3A**). However, rates of NO production were reduced by ∼half in two ABA treated *sitiens* plants (**Figure 5C**).

**FIGURE 6:**
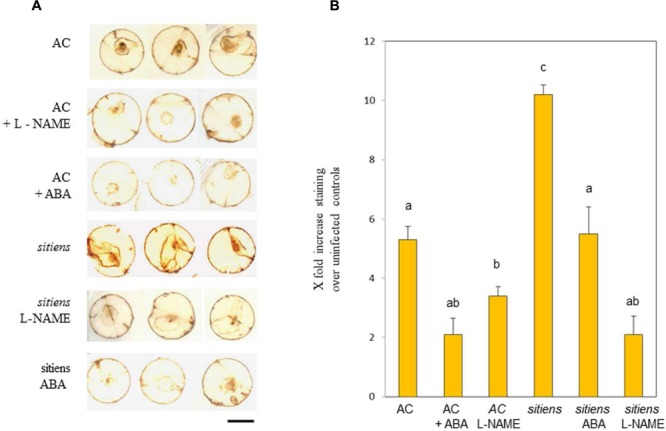
**Nitric oxide and abscisic acid (ABA) effects on *B. cinerea* elicited H_2_O_2_ production. (A)** H_2_O_2_ production as detected by staining with 3,3, diaminobenzidine (DAB) in 1 cm diameter representative cores from cv. Ailsa Craig (AC), *sitiens*, and *sitiens* sprayed with either 100 mM ABA (ABA) or 5 mM L-NAME at 24 h following inoculation with *B. cinerea.* **(B)** Quantification of DAB staining in 12 cores from each treatment specified by in **(A)**. Mean staining as indicated by Image Quant (±SE) are given. Significant differences at *P* < 0.05 are indicated.

In a seminal paper, Asselbergh et al., (2007) linked resistance in *sitiens* to elevated H_2_O_2_ production. We assessed if NO could also be contributing to H_2_O_2_ generation in cv. Ailsa Craig and *sitiens* in cores from tomato leaves at 24 h after inoculation with *B. cinerea* and stained with DAB (**Figure 6A**). Lesions with brown DAB staining were clearly observed in cv. Ailsa Craig and this appeared to be reduced by treatment with L-NAME and ABA. Greater staining was observed in *sitiens* compared to cv. Ailsa Craig and this was also reduced by L-NAME and ABA. This was further suggested when the extent of staining was measured using image analysis software (**Figure 6B**). Although a semi-quantitative measure at best, the data suggested significant reductions (*P* < 0.01) in DAB we seen in L-NAME/ABA treated cv. Ailsa Craig and *sitiens.* In the case of treatments of *sitiens* L-NAME/ABA treated plants results in patterns of DAB staining that were not significantly different from similarly treated cv Ailsa Craig samples. The data suggested that NO contributes to the elicitation of *B. cinerea* elicited H_2_O_2_ in wild type tomato and also to the elevated H_2_O_2_ observed in *sitiens.*

## Discussion

Nitric oxide effects on plant responses to stress have been extensive characterized using *Arabidopsis* but not exclusively so, with some important studies being undertaken in tomato, which has the virtue of being a both a model species and an important crop. Examples of studies establishing NO effects in stress tolerance in tomato include resistance to bacterial wilt (caused by *Ralstonia solanacearum*; Hong et al., 2013), root-knot nematode (*Meloidogyne incognita*; Zhou et al., 2015), Cu and Cd (Wang et al., 2015) and heat shock (Piterkova et al., 2013). Our study sought to investigate further investigate role of NO in the interaction of tomato with *B. cinerea.* Resistance to *B. cinerea* in has been shown to be influenced by Et (Robert-Seilaniantz et al., 2011) and we have demonstrated a link between NO and ethylene production in *B. cinerea*-challenged *Arabidopsis* (Mur et al., 2012). We now sought to employ our on-line, *in planta* measurement platforms based on QCL detection and photoacoustic laser detection (Mur et al., 2011) to develop our understanding of the pathogenic interaction. NO production had already been reported in tomato cultures in response to *B. cinerea* and correlated with ROS generation, PCD and activity of the *S*-nitrosoglutathione modulating enzyme, *S*-nitrosoglutathione reductase (GSNOR; Pietrowska et al., 2015). Also, use of SNP has been shown to increase resistance to *B. cinerea* via mechanisms that, at least in part, were influences by MAPKinases (Zheng et al., 2014).

Our initial experiments demonstrated very rapid production (within 30 min) of NO following challenge with *B. cinerea* (**Figure 1A**). These rapid induction kinetics are suggestive of responsiveness to PAMPs as described for chitosan from *Fusarium eumartii* (Terrile et al., 2015). Our use of L-NAME; (leaving aside doubts regarding its mechanism of action; Gupta et al., 2011) which may involve targeting NR (Rasul et al., 2012) suppressed NO production and lesion development establishing NO as a source of resistance in this pathosystem (**Figure 2**). How directly NO contributes to *B. cinerea* resistance needs to be clearly determined as L-NAME treated plants also displayed reduced Et production; an established mediator of defense against *B. cinerea* (**Figure 2F**).

In considering how defense hormones act, many workers focus on how these act either singly or via interaction to increase resistance to a given pathogen or pathogens with a given infection strategy (Robert-Seilaniantz et al., 2007, 2011). Equally, ABA signaling has been shown to be “hijacked” by a range of pathogens to increase virulence (Grant et al., 2013). Delivery of effectors is a key feature of pathogen-focused suppression of host defenses and some effectors appear to target ABA signaling. The AvrB,AvrC effector domain from *Xanthomonas campestris* pv. *campestris*-induced *NCED5*, encoding a key enzyme of ABA biosynthesis, and increased ABA was important in disease development by this pathogen (Ho et al., 2013). There is some evidence that AvrPtoB can fulfill a similar role (de Torres-Zabala et al., 2007). Over-expression of AvrPtoB (also known as HopAB2) in transgenic plants induced the ABA biosynthetic gene *NCED* and ABA signaling focusing on *abscisic acid insensitive 1* (*ABI1*) a protein phosphatases type 2C (PP2C). Alternatively, the *Pseudomonas syringae* effector HopAM1 enhances virulence via manipulation of sensitivity to ABA rather than initiating ABA biosynthesis (Goel et al., 2008). Moving beyond bacterial delivery of effectors, some fungal pathogen encode ABA biosynthetic genes and have been shown to synthesize ABA including *Cercopsora, Fusarium, Rhizoctonia* (reviewed by Cao et al., 2011), and of particular relevance to this current study, *B. cinerea* (Gong et al., 2014). Considering the mechanics of ABA promoted susceptibly, there is evidence that this causes reduced alterations in cell wall characteristics, ROS generation and callose deposition as well as defense gene expression (Asselbergh et al., 2007; de Torres-Zabala et al., 2007; Cao et al., 2011). Such wide ranging impact argues for multiple targets for ABA to confer increased susceptibility and paradigms from other systems; such as the phosphorylational regulation by ABA of the ROS generating NADPH oxidase AtrbohF in stomatal regulation, may be relevant (Sirichandra et al., 2009).

In order to provide a wider understanding of possible targets of ABA impacts on host responses we tested the possible interaction of ABA on NO effects. In undertaking this investigation we noted those few studies where ABA increases resistance (Ton and Mauch-Mani, 2004; Kaliff et al., 2007; De Vleesschauwer et al., 2010); thus as NO is key feature of both PTI and ETI (Mur et al., 2006) it was conceivable that ABA could increase NO generation. However, our analyses of the low ABA accumulating mutant *sitiens*, clearly indicated that ABA acted to suppress NO generation to promote virulence (**Figure 4**). This could implicate regulation of NR as a likely source of NO as a target for ABA manipulation. There is evidence for ABA acting via NR in *Arabidopsis* in the regulation of stomatal closure (Chen et al., 2015) and it is entirely conceivable that this is also the case in plant pathogen interactions. Thus, the regulation of NR by (for example) the phosphorylation of a conserved Ser residue or binding of 14-3-3 proteins could be targeted by ABA (Lillo et al., 2004). This stated it is important to unambiguously demonstrate that NR is the major source of NO in this *B. cinerea* – tomato interaction so that further work such as the use of RNAi or reduced NR activity lines is required. In this context, it should be noted that AtNOA1 although initially erroneously identified as a NO synthase (Guo et al., 2003), still contributes to NO –mediated events during plant responses to defense (Mandal et al., 2012). Indeed, although NOA1 was established as a mitochondria-located GTPase (Moreau et al., 2008) it appears to be regulating mitochondrial respiration (Heidler et al., 2011) which can be linked to NO production (Gas et al., 2009). Thus, modulation of NOA1 or mitochondrial function by ABA could be a target for ABA action. Indeed, ABA impact on certain mitochondrial GTPase and kinases (for example) have been noted (Zhao et al., 2015; Manara et al., 2016).

**FIGURE 7:**
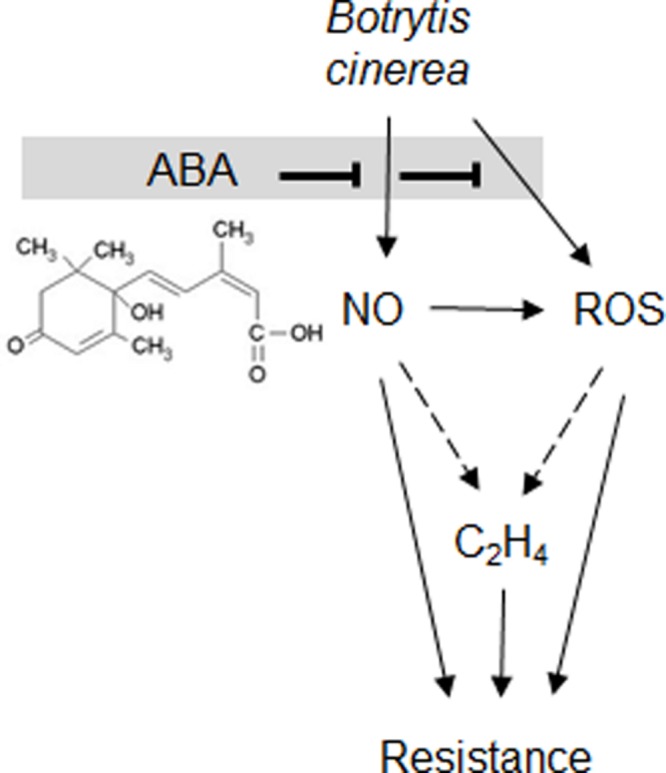
**Abscisic acid suppresses *B. cinerea* induced NO, H_2_O_2_ and ethylene production.** Based on our observations and the literature both NO and H_2_O_2_ are suppressed by ABA (chemical structure shown). This will impact on the hypothesized downstream elicitation of ethylene production (shown by dashed arrows) an established source of resistance against *B. cinerea.* Thus, the relative rates of ABA production by *B. cinerea* would represent an important virulence factor.

Increased resistance in ABA-deficient *sitiens* has been linked to elevated ROS production (Asselbergh et al., 2007) thus, the link between NO, ABA, and ROS was explored. Many studies have suggested that ROS generation is required to initiate NO production particularly in stomata (He et al., 2005; Lu et al., 2005; Bright et al., 2006; Shi et al., 2015). Additionally, ROS production was placed upstream of NO generation in the response of *Pisum sativum* to the PAMP, chitosan (Srivastava et al., 2009). Other authors have suggested that reduction of NO production through the use of NO scavengers or mutants resulted in increased ROS production (Tada et al., 2004; Asai et al., 2008) This could reflect a role for NO in modulating NADPH oxidase activity and this has been demonstrated by *S*-nitrosylation of AtrbohD (Asai et al., 2008; Yun et al., 2011). This could also reflect a role for NO as an ROS scavenger as suggested in early models (Beligni and Lamattina, 1999). In contrast, we demonstrated that the increase in ROS generation in response to *B. cinerea* in both *sitiens* and cv. Ailsa Craig could be reduced through the application of L-NAME (**Figure 6**). The placing of NO upstream of ROS generation was also suggested by Rasul et al. (2012) in oligogalacturonide-triggered immunity in *Arabidopsis*. Taken together, such studies would suggest that the relative positioning of ROS and NO is context specific; possibly even pathogen-interaction specific. Our placing of NO upstream of ROS could suggest that two major elicitory defense signals could be suppressed via a single target, i.e., NR-mediated NO production (**Figure 7**).

Considering our observations together, we have revealed a new node for pathogen-mediated suppression of host defenses, namely, NO generation. If NO is derived from NR activity, this would implicate either NR activity itself and/or $N_{3}^{–}$/$N_{2}^{–}$ assimilation as likely targets for ABA-mediated modulation. If this proves to be case, questions such as if the amount of available N or its form (Gupta et al., 2013) could influence the efficacy of ABA impacts on defense and therefore host resistance, become relevant.

## Author Contributions

AS, AA, JM, and LM produced the data used in this manuscript. AS carried out the estimations of fungal virulence based on fungal DNA content, H_2_O_2_ measurements, infections of different tomato genotypes. AA also contributed to estimations of fungal virulence based on fungal DNA content. JM and LM undertook the measurements of NO and ethylene. NO and ethylene measurements were supervised by SC and FH. Overall project supervision and direction was provided by LM. The manuscript was written by LM, FH, and SC.

## Conflict of Interest Statement

The authors declare that the research was conducted in the absence of any commercial or financial relationships that could be construed as a potential conflict of interest.

## Footnotes

1 http://tgrc.ucdavis.edu
